# Effects of Phage Cocktail, Probiotics, and Their Combination on Growth Performance and Gut Microbiota of Broiler Chickens

**DOI:** 10.3390/ani13081328

**Published:** 2023-04-13

**Authors:** Mohd Asrore Mohd Shaufi, Chin Chin Sieo, Chun Wie Chong, Tan Geok Hun, Abdul Rahman Omar, Gan Han Ming, Yin Wan Ho

**Affiliations:** 1 Department of Microbiology, Faculty of Biotechnology and Biomolecular Sciences, Universiti Putra Malaysia UPM, Seri Kembangan 43400, Malaysia; 2 Institute of Bioscience, Universiti Putra Malaysia UPM, Seri Kembangan 43400, Malaysia; 3 Department of Life Sciences, International Medical University, Jalan Jalil Perkasa 19, Taman Esplanade, Kuala Lumpur 57000, Malaysia; 4 Department of Land Management, Faculty of Agriculture, Universiti Putra Malaysia UPM, Seri Kembangan 43400, Malaysia; 5 School of Science, Monash University Malaysia, Jalan Lagoon Selatan, Bandar Sunway 47500, Malaysia

**Keywords:** antibiotic growth promoter, alternative, poultry production, next-generation sequencing, 16S rRNA, gut microbial diversity

## Abstract

**Simple Summary:**

The emergence of antibiotic-resistant bacteria and the growing demand for poultry products have led to an interest in finding alternatives to antibiotic growth promoters (AGPs) used in poultry farming. Probiotics, which have been shown to have positive effects on performance and health in chickens, are among the most recognised alternatives to AGPs. However, the use of probiotics in commercial farming has not been fully optimised. One of the major challenges arises from the competition of probiotics with other gut microbiota for adhesion and nutrients. This study investigated the use of a phage cocktail in combination with probiotics as a potential alternative to AGPs in poultry farming. The results showed that the combination of phage and probiotics improved growth performance in chickens and had a positive impact on the diversity and composition of gut microbiota. This study suggests that using a phage cocktail in combination with probiotics could be a promising alternative to AGPs for poultry production.

**Abstract:**

Phages, which are often used therapeutically, have begun to receive interest as alternatives to antibiotic growth promoters (AGPs) for enhancing chicken growth. Another option that has been extensively studied as a growth promoter in chickens is probiotics. To the best of our knowledge, there is currently no study available on the use of phages and probiotics in combination as potential feed additives for broiler chickens. Therefore, this study demonstrated the effects of a phage cocktail, probiotics, and their combination on the growth performance and gut microbiota of broiler chickens. A total of 288 one-day-old male Cobb 500 broilers were randomly allotted to one of six treatments in a completely randomised design. The treatments were (i) C (basal diet (BD) only), (ii) 1ϕ (BD + 0.1% phage cocktail), (iii) 2ϕ (BD + 0.2% phage cocktail), (iv) P (BD + 0.1% probiotic), (v) 1ϕP (BD + 0.1% phage cocktail + 0.1% probiotic), and (vi) 2ϕP (BD + 0.2% phage cocktail + 0.1% probiotic). The 1ϕP treatment had significantly (*p* < 0.05) better BW (35 days), BWG (22–35 days, 1–35 days), and FCR (1–21 days, 22–35 days, 1–35 days) compared to C. Unique gut microbiota diversity was also found between the ϕP (1ϕP and 2ϕP) and non-ϕP groups (C, 1ϕ, 2ϕ, and P) in ilea, particularly in the 35-day-old chickens. Microorganisms associated with short-chain fatty acid (SCFA) producers were significantly (*p* < 0.05) more present in the ϕP group than in the non-ϕP group. The predicted genes related to carbohydrate and amino acid metabolism were significantly upregulated in ϕP groups compared to non-ϕP groups. These genes were involved in the digestion and absorption of nutrients, as well as the production of energy. Our findings showed that the 1ϕP treatment could be a potential alternative to AGPs for poultry, as growth performance was enhanced, and gut microbiota was positively modulated.

## 1. Introduction

Poultry is the most important agricultural sector, providing the cheapest protein source to the global population. The use of antibiotic growth promoters (AGPs) in poultry production may be a significant factor that reduces production costs. AGPs are supplemented in feed at a subliminal amount, which can improve growth performance and reduce diseases in chickens [1,2,3]. The use of AGPs has been beneficial for animal production, but it has been reported to be one of the causes of antibiotic-resistant bacteria, resulting in serious health problems for humans due to the inefficacy of antibiotics to treat infections [4,5]. Hence, AGP use in livestock has been banned in certain parts of the world, such as European Union countries and South Korea [6,7,8]. The abolition of AGPs has affected the performance and health of broilers, necessitating the development of an effective substitute to provide the same level of sustainable chicken production as when AGPs are utilised [9,10]. Phage usage has, thus, begun to attract attention as a possible AGP replacement.

Phages are bacteria-infecting viruses that have recently attracted attention as an alternative to antibiotics. They have various advantages over antibiotics, including a narrow killing spectrum for targeting certain bacterial species, the ability to rapidly proliferate in the presence of a host and eventually kill the host, and easy isolation from the environment (Keen & Adhya, 2015; Rose et al., 2014; Ryan et al., 2011). Phages show similar mechanisms of action to AGPs, but the phage target is specific. Its application can reduce selected target bacterial species, thereby reducing competition with other normal gut microflora for adhesion sites and nutrient utilization. Phages are commonly employed for therapeutic purposes; however, they have recently been explored for gut modulation. Pathogens such as *Escherichia coli*, *Salmonella* spp., *Clostridium* spp., and coliforms were significantly reduced, and beneficial bacteria such as *Bifidobacterium* spp. and *Lactobacillus* spp. increased in pigs and chickens when phages were used to modulate the gut microbiota [11,12,13,14,15,16]. These studies indicate that phages could be utilised to modulate the gut microbiome to increase beneficial bacteria and decrease harmful bacteria. Probiotics, which are extensively studied as a potential AGP replacement, have never been used in conjunction with phage treatment in broiler chickens. Probiotics are live microorganisms that may benefit the host when administered sufficiently [17,18]. Previous studies have shown that probiotics improve the growth and health of broiler chickens [19,20,21,22].

In this study, the phage that targets non-pathogenic E. coli does not require thorough purification using caesium chloride (CsCl) and ultracentrifugation, in contrast to phages that target pathogens for therapeutic purposes [23,24]. In the case of phages that target non-pathogens, the bacterial debris present in the unpurified lysate may not provoke an immune response and kill the host. *E. coli* was also selected as the target bacterial host for the gut modification investigation because of its continuous presence in the ilea and caeca of broiler chickens at different ages [25,26]. Both *E. coli* host and phages were isolated previously from the ilea and caeca of healthy broiler chickens and characterised. Only non-pathogenic *E. coli* was selected for phage isolation, which was then used for further study. There is a paucity of knowledge about the impact of phages targeting non-pathogenic *E. coli*, probiotics, and their combination in chickens. Therefore, this study aimed to assess the effects of a phage cocktail that targets non-pathogenic *E. coli*, commercial probiotics, and their combination on the growth performance and gut microbiota of broiler chickens.

## 2. Materials and Methods

### 2.1. Probiotics and Phage Preparation

*Escherichia coli* P1, P2, P3, and P4 phages (prepared as a freeze-dried phage cocktail) at a titre of 10^10^ PFU/g each were prepared. Briefly, the carrier materials of skim milk (Oxoid, UK) for protecting phages from high temperature, along with industrial maltodextrin as a cryoprotectant and calcium carbonate (CaCO_3_) (Merck, Germany) as an antacid, were pre-dissolved with each diluted phage lysate at the ratio of 2:2:1:5, respectively. The phage lysate was diluted in sterile SM buffer (50 mM Tris-HCl [pH 7.5], 0.10 M NaCl, and 8 mM MgSO_4_·7H_2_O) to a final titre of ~1–5 × 10^10^ PFU/g. The mixture was then freeze-dried in a Labconco freeze-dryer (Labconco, Kansas City, MO, USA) by first pressing the vacuum when the temperature was at −40 °C or colder. Samples were then added when the vacuum was 1.33 × 10^−3^ mBar or lower. The titre of freeze-dried powder of each phage was quantified weekly until the week of the chicken trial. This was to ensure their survivability and concentration maintained at ~10^10^ PFU/g. The powder forms of each phage (C1, C2, C3, and C4 phages) were then mixed at the ratio of 1:1:1:1 to obtain a final phage cocktail form to be supplemented into the chicken feeds. Commercial PrimaLac^®^ probiotic (Starlabs, Piscataway, NJ, USA) used in this study consisted of *Lactobacillus acidophilus*, *Lactobacillus casei*, *Bifidobacterium termophilum*, *Enterococcus faecium*, and *Aspergillus oryzae* at a concentration of 10^9^ CFU/g each. The phage cocktail and commercial probiotic were mixed with basal feed fresh daily before being fed to the chickens.

### 2.2. Chicken Management

A total of 288 1 day old male Cobb 500 broiler chickens (initial body weight (BW) = 42.95 ± 2.26 g) were obtained from a local commercial hatchery. They were housed in stainless-steel three-tiered battery cages with raised wire floors (dimensions: 116 cm width × 89 cm length × 46 cm height) in an open-house facility at the Animal Research Centre (ARC), Institute of Tropical Agriculture, Universiti Putra Malaysia, Malaysia. The cages were cleaned and disinfected through fumigation beforehand, and strict hygiene and biosecurity measures were practiced throughout the experiment. The feeders and drinkers were cleaned and filled with fresh feed and water daily. The temperature and relative humidity were recorded twice daily in the morning and the afternoon. For the chicken brooding period, lasting for the first 14 days, lighting from a 100 W bulb per cage was provided for each replicate cage. The raised wire floors were covered with newspaper and cleaned daily. Procedures pertaining to chicken management, experimental design, and analyses were approved by the Institutional Animal Care and Use Committee (IACUC) Universiti Putra Malaysia (Ref: UPM/IACUC/AUP-R101/2015).

### 2.3. Experimental Design, Animals, and Diets

A total of 288 1 day old male Cobb 500 broilers were randomly allotted to one of six dietary treatments with six replicate cages containing eight chicks per cage in a completely randomised design. The dietary treatments were (i) basal diet only (BD) (control, C), (ii) BD + 1 g/kg phage cocktail (1ϕ), (iii) BD + 2 g/kg phage cocktail (2ϕ), (iv) BD + 1 g/kg probiotic (P), (v) BD + 1 g/kg phage cocktail + 1 g/kg probiotic (1ϕP), and (vi) BD + 2 g/kg phage cocktail + 1 g/kg probiotic (2ϕP). Both the phage cocktail and the probiotics for respective treatments were supplemented at the expense of corn to achieve a final equal percentage for each treatment group. The basal diets formulated for starter (1 to 21 days) (Table 1) and finisher (22 to 35 days) (Table 2) periods were antibiotic-free, in mash form, meeting or exceeding the energy and nutrient requirements as recommended by the NRC [27] for each growing phase. Both feeds and water were provided ad libitum.

### 2.4. Chemical Analysis of Experimental Diets

The formulated starter feed, finisher feed, phage cocktail, and probiotic were chemically analysed through feed proximate analyses (Malaysian Agricultural Research and Development Institute (MARDI)) on crude protein, crude fat, crude fibre, calcium, phosphorus, and sodium (Table 1 and Table 2).

### 2.5. Sampling

For every sampling period on day 21 and 35, 12 chickens per treatment (two chickens per replicate cage) were randomly selected, weighed, and euthanised by severing the jugular veins. The mucosal contents of ilea and caeca samples were used for the gut microbiota study based on high-throughput next-generation sequencing (HT-NGS) of 16S rRNA gene amplicons. All samples were kept on ice before the respective samples were processed. They were then frozen at −80 °C until analysis.

### 2.6. Growth Performance

The chicken body weight (BW) and body weight gain (BWG) were individually recorded on a weekly basis (at 1, 7, 14, 21, 28, and 35 days), while feed intake (FI) and replicate cages were recorded daily. The mortality rate was checked and recorded daily, while the number of chickens for FI calculation was then adjusted accordingly. The body weight gain (BWG), feed conversion ratio (FCR), and mortality rate were calculated as described by Naidoo, et al. [28] and Wang and Xu [29]:

$$\mathrm{Body}\;\mathrm{weight}\;\mathrm{gain}\;\left(BWG\right)=\frac{Final\;body\;weight-Initial\;body\;weight}{Number\;of\;birds}.$$

$$\mathrm{Feed}\;\mathrm{conversion}\;\mathrm{ratio}\;(\mathrm{FCR})=\frac{Feed\;intake\;(FI)}{BWG}.$$

$$\mathrm{Mortality}\;\mathrm{rate}=\frac{\mathrm{Number}\;\mathrm{of}\;\mathrm{dead}\;\mathrm{birds}\;\mathrm{in}\;\mathrm{treatment}\;\mathrm{group}}{\mathrm{Number}\;\mathrm{of}\;\mathrm{initial}\;\mathrm{birds}\;\mathrm{in}\;\mathrm{each}\;\mathrm{treatment}\;\mathrm{group}}\times 100.$$

### 2.7. Chicken Gut Microbiota Study

#### 2.7.1. DNA Extraction

Genomic DNA was extracted using a QIAamp Fast DNA Stool Mini Kit (QIAGEN, Stockach, Germany). The mucosal contents of the ilea and caeca (~180–220 mg) were treated with lysozyme lysis buffer (25 mg/mL lysozyme; 20 mM Tris-Cl, pH 8.0; 2 mM EDTA, pH 8.0; 1% Triton X-100) and incubated for 30 min at 37 °C to facilitate the lysis of Gram-positive bacteria. Then, 1 mL of InhibitEX Buffer was added to each sample and vortexed continuously for 1 min. The mixtures were then heated for 5 min at 95 °C and vortexed for 10 s to lyse Gram-positive bacteria. Subsequently, the solution was centrifuged at 16,100× *g* for 1 min to pellet the intestinal debris. The supernatants were treated with 4 µL of 5 µg/mL RNase A (Epicentre, WI, USA) and incubated for 30 min at 37 °C to remove RNA. The eluted genomic DNA was then stored at −20 °C before further use.

#### 2.7.2. Illumina Sequencing of the V3–V4 Region of the 16S rRNA Gene

The V3–V4 hypervariable region of 16S rRNA gene was amplified using the forward primer (5′–TCGTCGGCAGCGTCAGATGTGTATAAGAGACAGNNNNCCTACGGGNG GCWGCAG–3′) and reverse primer (5′–GTCTCGTGGGCTATAAGAGACA GGACTACHVGGGTATCTAATCC–3′) (Integrated DNA Technologies (IDT), Singapore) as described by Klindworth, et al. [30], with some modifications. Four degenerate bases (N) were added to maximise the diversity for unique cluster identification. The purified amplicons were quantified using a Qubit Fluorometer (ThermoFisher Scientific, Waltham, MA, USA), normalised to 2 nM, and subjected to Illumina Miseq desktop sequencing using paired 300 bp reads with the Miseq Reagent Kit v3 (600-cycle) (Illumina, CA, USA) at Monash University Malaysia Genomic Facility.

#### 2.7.3. Bioinformatics Analysis

Sequences in FASTQ format were assembled and quality-filtered using the Mothur software package (v. 1.38.1) [31]. The processed sequences were subsampled to 9998 prior to alpha diversity analyses. The table of raw OTUs was normalised using the “cumNorm” command. In addition, OTUs with significant differences in abundance were selected on the basis of a zero-inflated log-normal model using the “fitFeatureModel” command. Both “cumNorm” and “fitFeatureModel” were implemented in metagenomSeq package [32]. The table of normalised OTUs was exported into PRIMER7 with the PERMANOVA add-on programme package (Plymouth Marine Laboratory, Plymouth, UK) and converted to the Bray–Curtis similarity index for group comparison [i.e., permutational multivariate analysis of variance (PERMANOVA)] and statistical ordinations [i.e., canonical analysis of principal coordinates (CAP) and principal coordinate analysis (PCO)].

#### 2.7.4. Microbial Predicted Functional Metagenomes Based on PICRUSt and STAMP

The prediction of metagenome function from the 16S rRNA marker gene was performed using Phylogenetic Investigation of Communities by Reconstruction of Unobserved States (PICRUSt) version 1.1.0 [33]. The Greengenes-based biom file was generated using Mothur software and uploaded into online galaxy PICRUSt (http://huttenhower.sph.harvard.edu/galaxy/ (accessed on 13 March 2023)). The OTU table was first normalised for multiple 16S copy numbers, where the genome was then predicted according to Kyoto Encyclopaedia of Genes and Genomes (KEGG) ortholog abundances. The output files were then analysed using the Statistical Analysis of Metagenomic Profiles (STAMP) version 2.1.3 bioinformatics software package [34]. Storey’s FDR multiple test correction method was used to calculate the statistical significance, and the features were filtered by an effect size at 0.05.

#### 2.7.5. Nucleotide Sequence Accession Numbers

The V3–V4 regions of 16S rRNA gene sequences from this study were deposited in the NCBI sequence read archive (https://www.ncbi.nlm.nih.gov/sra (accessed on 13 March 2023)) under BioSample Accession numbers SAMN06027949–SAMN06028092.

### 2.8. Statistical Analysis

The experimental data were analysed with a one-way analysis of variance (ANOVA) and paired *t*-test using the Statistical Package for Social Science (SPSS) Statistics version 22 (IBM, New York, NY, USA). The replicate cage was used as the experimental unit for all parameters, unless stated otherwise. Results were stated as means or means ± standard error (SE). Significant means with *p* < 0.05 were compared within samples by Duncan’s multiple range test [35].

## 3. Results

### 3.1. Growth Performance

The effects of dietary treatments on growth performance are presented in Table 3. At 21 days of age, there were no significant differences in BW or BWG among the treatments (Table 3). After birds were selected and removed for sampling, there were still no differences among the treatments in these parameters, according to the remaining birds moving forward in the experiment (data not shown). Chickens fed with 1ϕP had significantly (*p* < 0.05) better BW (35 days), BWG (22–35 days, 1–35 days), and FCR (1–21 days, 22–35 days, 1–35 days) compared to the control. However, this was not the case for chickens fed with P, where the FCR was not significantly (*p* > 0.05) different compared to the control in the period of 1–21 days. The phage cocktail alone (1ϕ and 2ϕ) showed significantly better (*p* < 0.05) FCR (1–21 days, 22–35 days, 1–35 days) compared to the control group. The highest mortality rate was recorded in both the control and the 2ϕ groups at 4.17%, whereas no mortality was observed in the P group.

### 3.2. Multivariate Analysis on Gut Microbiota

The study employed a principal coordinate analysis (PCO) plot of the Bray-Curtis similarity index to investigate the structure of the gut microbiota. The analysis considered dietary treatment, age (21 and 35 days), and part of the intestine (ilea and caeca). The results showed a clear separation of chicken gut microbiota based on age (21 and 35 d) (Figure S2) and part of intestine (ilea and caeca) (Figure S3) was observed. Additionally, the ϕP (1ϕP and 2ϕP) gut microbiota was significantly different from the non-ϕP group (C, 1ϕ, 2ϕ, and P) (Figure S4).

**Figure 1:**
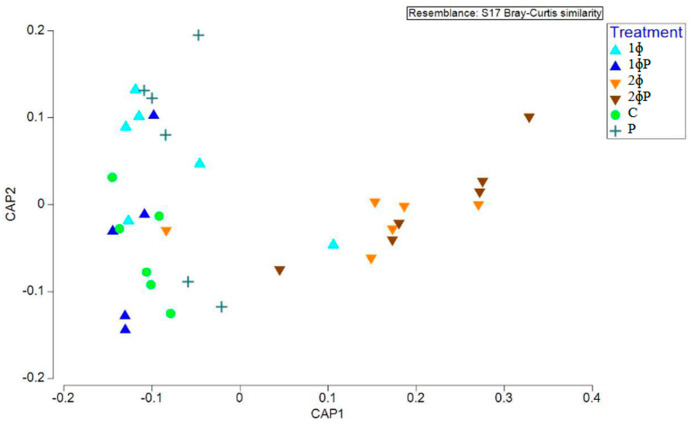
Structure of gut microbiota supplemented with different dietary treatments in ilea of 35 day old chickens investigated using the canonical analysis of principal coordinates (CAP) of the Bray–Curtis similarity index. Treatments: C = control (basal diet); 1ϕ = BD + 1 g/kg phage cocktail; 2ϕ = BD + 2 g/kg phage cocktail; P = BD + 1 g/kg probiotic; 1ϕP =BD + 1 g/kg phage cocktail + 1 g/kg probiotic; 2ϕP = BD + 2 g/kg phage cocktail + 1 g/kg probiotic.

Further analysis was conducted using the canonical analysis of principal coordinates (CAP) plot to investigate differences in the gut microbiota structure among the dietary treatments. The CAP plot revealed distinct differences between the ϕP gut microbiota and the non-ϕP group, particularly in the ilea of 21- and 35-day-old chickens (Figure S5), as well as in the ilea of 35-day-old chickens. (Figure 1).

Further verifications were performed to validate the earlier patterns observed from the PCA plot. The hypothesis test on the difference in gut microbiota according to age, part of the intestine, and dietary treatment was verified using the permutational multivariate analysis of variance (PERMANOVA) marginal test.

According to the PERMANOVA marginal test, the diversity of gut microbiota was found to be significantly different based on age (*p* = 0.002) (Table S2), part of intestine (*p* = 0.001) (Table S2), dietary treatment in the ilea (*p* = 0.005) (Table S3), and dietary treatment in the ilea of 35 day old chickens (*p* = 0.001) (Table 4). The patterns observed earlier in a CAP plot were further verified by the PERMANOVA pairwise test, which was conducted based on dietary treatment in the ilea of 21 and 35-day-old chickens (Table S3b) as well as the ilea of 35-day-old chickens (Table 4b). These tests revealed a significant difference in gut microbiota between the ϕP and non-ϕP groups. 

### 3.3. Significant OTUs Present in the ϕP Compared to the Non-ϕP Groups

The OTUs that were significantly expressed in the phage cocktail and probiotic combination groups (ϕP) were identified using “fitFeatureModel” in the metagenomeSeq package (Table 5). The value of logFC is directly related to abundance in the ϕP groups. Of the top 50 OTUs selected, the most common bacterial genera or species that were significantly elevated in ϕP groups, compared to non-ϕP groups, were *Bacteroides, Odoribacter*, *Alistipes*, *Anaerotruncus*, *Ruminococcaceae*, *Lachnospiraceae*, *Ruminococcus*, *Desulfovibrio*, *Anaerostipes*, *Clostridium*, *Coprobacillus*, *Butyricimonas*, *Faecalibacterium prausnitzii*, and *Oscillopira*.

### 3.4. Microbial Predicted Functional Metagenomes

**Figure 2:**
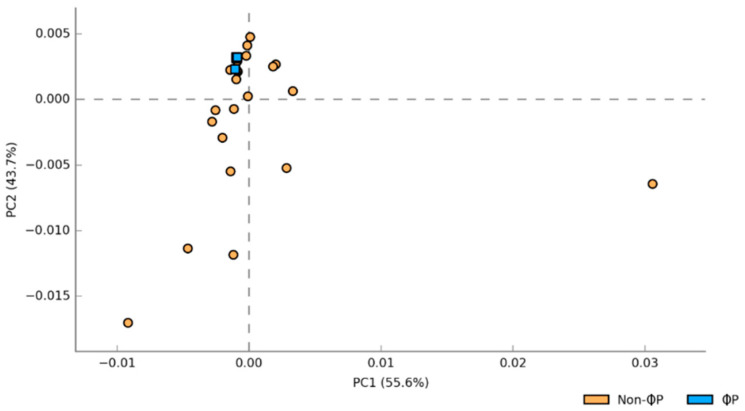
Principal component analysis (PCA) of predicted functional metagenomes based on ϕP versus non-ϕP groups. Phage cocktail and probiotic combination groups (ϕP): 1ϕP and 2ϕP; other groups (non-ϕP): C, 1ϕ, 2ϕ, and P.

**Figure 3:**
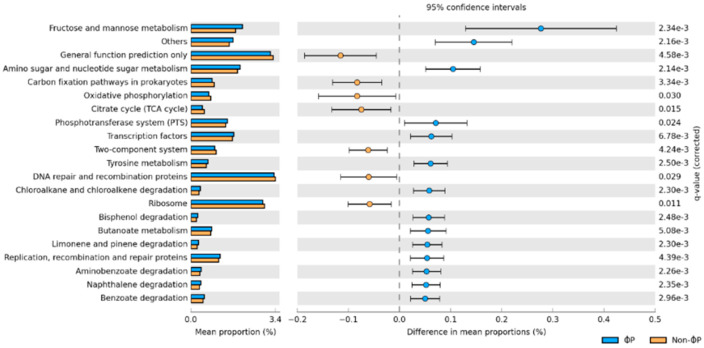
Pairwise comparison of the functional metagenomes predicted from Storey’s FDR multiple test correction methods based on ϕP versus non-ϕP groups. Phage cocktail and probiotic combination groups (ϕP): 1ϕP and 2ϕP; other groups (non-ϕP): C, 1ϕ, 2ϕ, and P.

The gut microbiota was unique between ϕP and non-ϕP groups according to the functional metagenomes predicted by STAMP analysis (Figure 2). There were also significant differences in metabolic pathways between ϕP and non-ϕP groups according to functional metagenomes predicted from filtered Storey’s FDR multiple test correction analyses (Figure 3). Fourteen out of 21 KEGG features were significantly higher in ϕP compared to non-ϕP groups. The metabolic pathways of ϕP groups related to carbohydrate (e.g., fructose and mannose metabolism), amino-acid (e.g., amino sugar and nucleotide sugar metabolism), and tyrosine metabolism were significantly elevated compared to those of non-ϕP groups.

## 4. Discussion

This is the first study to investigate the effects of dietary supplementation with freeze-dried *Escherichia coli* phage cocktail at different dosages (four different phages that lyse four different *E. coli* strains at 10^10^ PFU/g each), commercial probiotics (*Lactobacillus acidophilus*, *Lactobacillus casei*, *Bifidobacterium termophilum*, *Enterococcus faecium*, and *Aspergillus oryzae* at 10^9^ CFU/g each), and their combination on the growth performance and gut microbiota diversity of broiler chickens. The experiment was performed in normal physiological conditions without bacterial (*E. coli*) challenge. The present study also demonstrates, for the first time, the use of a phage targeting non-pathogenic *E. coli*. This study showed that supplementing chickens with a combination of phage cocktail and probiotics may have positively influenced growth performance and modulated the gut microbiota.

Growth performance parameters are the most important parameters to evaluate the efficacy of feed supplements in broiler production. High BW, BWG, FI, and low FCR indicate improved growth performance. The results of the present study showed that the growth performance of chickens fed with 1ϕP was significantly (*p* < 0.05) better than control (Table 3); 1ϕP also showed better FCR (1–21 days) compared to control than the P group. The group that received a higher dosage of the 2ϕP phage did not exhibit improved growth performance compared to the group that received 1ϕP, indicating a limit to the benefits of phage, and suggesting that increasing the dosage beyond a certain point does not provide any additional benefits. No previous study investigated the effects of a combination of phages and probiotics in chicken, hindering comparison. However, studies in pigs have shown that supplementation with 1 g/kg and 1.5 g/kg commercial phage cocktail (*Salmonella typhimurium*, *Salmonella enteritidis*, *Salmonella cholerasuis*, and *Salmonella derby*, *Staphylococcus aureus*, *Escherichia coli* (k88, k99, and f41), and *Clostridium perfringens* types A and C at 10^9^ PFU/g each) without bacterial challenge and in combination with 3 g/kg probiotics (*Lactobacillus acidophilus K31*, *Bacillus subtilis K 42*, and *Saccharomyces cerevisiae K47*) at 10^8^, 10^9^, and 10^4^ CFU/g, respectively) did not lead to any significant improvement in growth performance compared to phage alone (Kim et al., 2016). This contrasts the findings reported here because pigs and broilers have distinct physiological gut environments.

In the current study, 1ϕ and 2ϕ groups showed significantly (*p* < 0.05) better FCR (1–21 days, 22–35 days, 1–35 days) than the control, in accordance with other studies. A previous study demonstrated that the FCR of broilers supplemented with 1 mL of 10^10^ PFU/mL *Salmonella typhimurium* phage with the bacterial challenge was significantly better than that of the control [16]. This result is in contrast to the findings of Upadhaya, et al. [36], who found that broilers supplemented with 0.5 g/kg and 1 g/kg phage cocktail (*S. gallinarum*, *S. typhimurium*, *S. enteritidis*, and *E. coli* at 1.0 × 10^8^ PFU/g each, and *C. perfringens* at 1.0 × 10^6^ PFU/g) without bacterial challenge only showed slightly (not significant) better BWG, FI, and FCR than the control. In our study, there was no difference in growth performance between low (1 g/kg of 10^10^ PFU/g) and high (2 g/kg of 10^10^ PFU/g) dosages of phages. Our result is in contrast with a study by Wang, Yan, Lee and Kim [14], who reported that a higher dosage of 0.5 g/kg *S. typhimurium* phage (10^8^ PFU/g) without bacterial challenge showed significantly better FCR (1–14 days) compared to control than the lower dosage of 0.25 g/kg *S. typhimurium* phage (10^8^ PFU/g). This inconsistency may be because the concentration of phage used in our study was already very high at 10^10^ PFU/g compared to others at 10^8^ PFU/g. A note of caution is also due here since previous studies mentioned earlier incorporated phages targeting pathogens such as *S. typhimurium*, *S. gallinarum*, *S. enteritidis*, *C. perfringens*, and *E. coli.* It is evidenced from this study that phage supplements that target non-pathogenic *E. coli* could also result in a significant improvement in growth performance.

According to the CAP plot and PERMANOVA pairwise test, a distinct gut microbiota was found between the ϕP (1ϕP and 2ϕP) and non-ϕP groups (C, 1ϕ, 2ϕ, and P) in the ilea of 35 day old chickens. No prior research has examined the effects of phages, probiotics, and their combination on the diversity of the gut microbiota based on 16S rRNA HT-NGS, hindering comparison. Upadhaya, Ahn, Cho, Kim, Kang, Kim, Kim and Kim [36], however, demonstrated that chickens supplied with 0.5 g/kg phage cocktail, 1 g/kg phage cocktail, and 0.25 g/kg Avilamix (antibiotic) had distinctively different gut microbiota according to unweighted UniFrac. This demonstrates that the diversity of the gut microbiota was altered by phage supplementation in broiler chickens. On the other note, in accordance with earlier studies, the chicken gut microbiota showed distinct differences based on age (*p* = 0.002) (21 days and 35 days) and section of the intestine (*p* = 0.001) (ilea and caeca) [25,37,38,39].

In the current study, the OTUs that were significantly (*p* < 0.05) present in the ϕP with respect to the non-ϕP groups were related to short-chain fatty acid (SCFA) producers (e.g., *Bacteroides*, *Odoribacter*, *Alistipes*, *Anaerotruncus*, *Ruminococcus*, *Clostridiales*, *Clostridium*, *Desulfovibrio*, *Butyricimonas*, *Faecalibacterium prausnitzii*, *Anaerostipes*, and *Phascolarctobacterium*). These SCFA producers have known roles for excreting various enzymes to facilitate the breakdown of non-starch polysaccharides (NSP) to SCFAs such as acetic, succinic, propionic, and butyric acid in chickens [40,41,42,43]. The presence of these SCFA producers is associated with better digestion and energy production that improved growth performance in chickens [44]. The SCFAs produced from the breakdown and fermentation of polysaccharides (e.g., cellulose and hemicellulose) have also been recognised as an important source of energy for the host. Furthermore, SCFA producers have also been reported to inhibit the growth of pathogens such as *Salmonella* spp. and *C. perfringens* according to the reduction of intestinal pH, excretion of mucin, and host antimicrobial peptides [43,45,46,47]. The beneficial effects reported on the presence of SCFA producers could have been responsible for the improvement of chicken growth performance in the ϕP groups, especially the 1ϕP group. However, the roles of phages and probiotic combinations in promoting SCFA producers were not clear.

Based on the microbial predicted metagenome study, genes related to nutrient digestion and absorption and energy production, such as carbohydrate metabolism (fructose and mannose metabolism; butanoate metabolism) and amino-acid metabolism (amino sugar and nucleotide sugar metabolism) were significantly upregulated in ϕP compared to non-ϕP groups. These findings are consistent with our earlier findings that SCFA producers significantly present in ϕP groups could be capable of hydrolysing carbohydrates such as non-starch polysaccharides (NSPs) (e.g., glucose, fructose, starch, and fructooligosaccharide) [48]. Another predicted function is the phosphotransferase system (PTS). The PTS has been demonstrated to facilitate the nutrient uptake of carbohydrate, glycerol, and phosphate in members of *Firmicutes* bacteria [42]. The higher microbial predicted function related to metabolism and nutrient absorption suggests that ϕP groups gut microbiota were modulated, which may have resulted in the improvement in chicken growth performance in the 1ϕP supplemented group, especially in the period of 1–21 days. However, these predicted microbial metagenome data need to be interpreted with caution as they can only be accurately assigned to the sequences present in the database, whereas there is no assignment available for novel bacteria [49]. In addition, the predicted genes are not necessarily expressed in the host. Therefore, a further validation step using targeted reverse transcriptase or shotgun metagenomics is required to study the RNA expression of target genes.

It is unclear exactly how phages and probiotics affected the production of these SCFAs in the gut microbiota, which may have also positively influenced growth performance. Further research is required to understand the mechanisms underlying the efficacy of phage and probiotic combinations in enhancing growth performance and gut microbiota diversity. It was proposed that the synergistic effects of the *E. coli* phage cocktail and probiotic combination may have supplied additional adhesion sites and nutrients, as well as lowered toxins that favour the colonisation of these SCFAs producers. Although the advent of 16S rRNA of HT-NGS provides an unprecedented depth of sequencing gut microbiota, it unable to differentiate between viable and nonviable microorganisms [50]. Knowing whether or not a microorganism is viable is important, particularly in research utilising phages. Future studies could focus on treating DNA samples with propidium monoazide (PMA), which is able to distinguish the viability of microorganisms [51].

## 5. Conclusions

This study was carried out to determine the effects of an *E. coli* phage cocktail, commercial probiotics, and their combination on the growth performance and modulation of gut microbiota in the ilea and caeca of 21 and 35 day old broiler chicken. Specifically, this study identified that supplementing a combination of 1 g/kg phage cocktail and probiotic (1ϕP) to broiler chickens significantly improved chicken growth performance and positively modulated gut microbiota. The 1ϕP group also had significantly better FCR (1–21 days) compared to the control than the probiotic (P) group. The OTUs related to SCFAs producers were dominantly observed in ϕP compared to the non-ϕP groups. They might be responsible for gut microbiota modulation that facilitates carbohydrate and amino-acid metabolism, as well as nutrient uptake, thereby providing energy for chicken growth. The predicted microbial metagenomes also showed that the genes related to carbohydrate and amino-acid metabolism and nutrient uptake were significantly present in ϕP compared to the non-ϕP groups. The results indicates that the 1ϕP supplement could be considered a potential alternative to AGPs for poultry.

## Figures and Tables

**Table 1:** Ingredient composition, calculated analysis and chemical analysis of basal diet’s starter phase (1 to 21 days).

	Basal Diet (C)	BD + 1 g/kg Phage Cocktail (1ϕ)	BD + 2 g/kg Phage Cocktail (2ϕ)	BD + 1 g/kg Probiotic (P)	BD + 1 g/kg Phage Cocktail + 1 g/kg Probiotic (1ϕP)	BD + 2 g/kg Phage Cocktail + 1 g/kg Probiotic (2ϕP)
Ingredient (g/kg)						
Corn	538.60	537.60	536.60	537.60	536.60	535.60
Soybean meal, 48% Cp	361.90	361.90	361.90	361.90	361.90	361.90
Fish meal	30.00	30.00	30.00	30.00	30.00	30.00
Palm oil	37.40	37.40	37.40	37.40	37.40	37.40
60% choline chloride	2.50	2.50	2.50	2.50	2.50	2.50
Vitamin premix ^†^	0.30	0.30	0.30	0.30	0.30	0.30
Mineral premix ^‡^	1.00	1.00	1.00	1.00	1.00	1.00
Salt (NaCl)	2.00	2.00	2.00	2.00	2.00	2.00
DL-Methionine	1.80	1.80	1.80	1.80	1.80	1.80
Limestone	13.00	13.00	13.00	13.00	13.00	13.00
Dicalcium phosphate	11.50	11.50	11.50	11.50	11.50	11.50
Phage cocktail	0.00	1.00	2.00	0.00	1.00	2.00
Probiotic	0.00	0.00	0.00	1.00	1.00	1.00
Total	1000.00	1000.00	1000.00	1000.00	1000.00	1000.00
Calculated analysis						
Metabolisable energy (MJ/kg)	13.06	13.06	13.06	13.06	13.06	13.06
Crude protein	220.00	220.00	220.00	220.00	220.00	220.00
Crude fat	63.10	63.10	63.10	63.10	63.10	63.10
Crude fibre	38.00	38.00	38.00	38.00	38.00	38.00
Digestible methionine + cysteine	9.50	9.50	9.50	9.50	9.50	9.50
Digestible lysine	13.70	13.70	13.70	13.70	13.70	13.70
Calcium	10.20	10.20	10.20	10.20	10.20	10.20
Available phosphorus	4.50	4.50	4.50	4.50	4.50	4.50
Chemical analysis						
Crude protein	217.70	217.70	217.70	217.70	217.70	217.70
Crude fat	54.80	54.80	54.80	54.80	54.80	54.80
Crude fibre	38.80	38.80	38.80	39.01	39.02	39.02
Calcium	6.50	6.55	6.61	6.66	6.71	6.77
Phosphorus	5.16	5.16	5.16	5.16	5.16	5.16
Sodium	6.70	6.71	6.72	6.70	6.71	6.72

^†^ Supplied per kilogram of diet: vitamin A, 50.00 mIU; vitamin B1, 10.00 g; vitamin B2, 30.00 g; vitamin B6, 20.00 g; vitamin B12, 0.100 g; vitamin D3, 10.00 mIU; vitamin E, 75.00 g; vitamin K3, 20.00 g; calcium D-pantothenate, 60.00 g; nicotinic acid, 200.00 g; folic acid, 5.00 g; biotin, 235.00 g; antioxidant, anti-cracking, and carrier. ^‡^ Supplied per kilogram of diet: Se, 0.200 g; Fe, 80.00 g; Mn, 100.00 g; Zn, 80.00 g; Cu, 15.00 g; KCl, 4.00 g; MgO, 0.60 g; NaCO_3_, 1.50 g; I, 1.00 g; Co, 0.25 g.

**Table 2:** Ingredient composition, calculated analysis, and chemical analysis of basal diet’s finisher phase (22 to 35 days).

	Basal Diet (C)	BD + 1 g/kg Phage Cocktail (1ϕ)	BD + 2 g/kg Phage Cocktail (2ϕ)	BD + 1 g/kg Probiotic (P)	BD + 1 g/kg Phage Cocktail + 1 g/kg Probiotic (1ϕP)	BD + 2 g/kg Phage Cocktail + 1 g/kg Probiotic (2ϕP)
Ingredient (g/kg)						
Corn	602.70	601.70	600.70	601.70	600.70	599.70
Soybean meal, 48% Cp	318.60	318.60	318.60	318.60	318.60	318.60
Fish meal	30.00	30.00	30.00	30.00	30.00	30.00
Palm Oil	24.50	24.50	24.50	24.50	24.50	24.50
60% choline chloride	2.00	2.00	2.00	2.00	2.00	2.00
Vitamin premix ^†^	0.30	0.30	0.30	0.30	0.30	0.30
Mineral premix ^‡^	1.00	1.00	1.00	1.00	1.00	1.00
Salt (NaCl)	1.00	1.00	1.00	1.00	1.00	1.00
DL-Methionine	0.40	0.40	0.40	0.40	0.40	0.40
Limestone	13.00	13.00	13.00	13.00	13.00	13.00
Dicalcium phosphate	6.50	6.50	6.50	6.50	6.50	6.50
Phage cocktail	0.00	1.00	2.00	0.00	1.00	2.00
Probiotic	0.00	0.00	0.00	1.00	1.00	1.00
Total	1000.00	1000.00	1000.00	1000.00	1000.00	1000.00
Calculated analysis						
Metabolisable energy (MJ/kg)	13.06	13.06	13.06	13.06	13.06	13.06
Crude protein	199.90	199.90	199.90	199.90	199.90	199.90
Crude fat	52.20	52.20	52.20	52.20	52.20	52.20
Crude fibre	36.50	36.50	36.50	36.50	36.50	36.50
Digestible methionine + cysteine	8.50	8.50	8.50	8.50	8.50	8.50
Digestible lysine	12.00	12.00	12.00	12.00	12.00	12.00
Calcium	9.00	9.00	9.00	9.00	9.00	9.00
Available phosphorus	3.50	3.50	3.50	3.50	3.50	3.50
Chemical analysis						
Crude protein	212.05	212.50	212.50	212.50	212.50	212.50
Crude fat	45.00	45.00	45.00	45.00	45.00	45.00
Crude fibre	40.50	40.50	40.50	40.71	40.71	40.71
Calcium	6.80	6.85	6.91	6.96	7.01	7.07
Phosphorus	4.50	4.50	4.50	4.50	4.50	4.50
Sodium	1.90	1.91	1.92	1.90	1.91	1.92

^†^ Supplied per kilogram of diet: vitamin A, 50.00 MIU; vitamin B1, 10.00 g; vitamin B2, 30.00 g; vitamin B6, 20.00 g; vitamin B12, 0.100 g; vitamin D3, 10.00 MIU; vitamin E, 75.00 g; vitamin K3, 20.00 g; calcium D-pantothenate, 60.00 g; nicotinic acid, 200.00 g; folic acid, 5.00 g; biotin, 235.00 g; antioxidant, anti-cracking, and carrier. ^‡^ Supplied per kilogram of diet: Se, 0.200 g; Fe, 80.00 g; Mn, 100.00 g; Zn, 80.00 g; Cu, 15.00 g; KCl, 4.00 g; MgO, 0.60 g; NaCO_3_, 1.50 g; I, 1.00 g; Co, 0.25 g.

**Table 3:** Effects of phage cocktail, probiotics, and their combination on growth performance of chickens.

Item ^†^	Age (days)	C	1ϕ	2ϕ	P	1ϕP	2ϕP
BW (g)	1	43.00 ± 1.10	42.78 ± 1.09	42.98 ± 0.78	42.82 ± 0.96	42.78 ± 1.05	43.32 ± 0.38
	21	859.58 ± 14.20	874.07 ± 8.85	864.67 ± 11.05	871.78 ± 6.09	874.02 ± 12.31	855.10 ± 13.74
	35	1605.30 ± 39.45 ^a^	1734.17 ± 47.55 ^ab^	1741.27 ± 56.58 ^ab^	1690.43 ± 40.05 ^ab^	1785.75 ± 31.93 ^b^	1733.63 ± 56.00 ^ab^
BWG (g/bird)	1–21	816.63 ± 13.97	861.32 ± 9.47	821.70 ± 11.09	828.98 ± 6.04	831.25 ± 11.91	811.80 ± 12.95
	22–35	745.72 ± 40.37 ^a^	860.10 ± 50.69 ^ab^	876.60 ± 46.20 ^ab^	818.65 ± 41.87 ^ab^	911.73 ± 32.82 ^b^	878.53 ± 47.67 ^ab^
	1–35	1562.35 ± 40.42 ^a^	1691.42 ± 47.67 ^ab^	1698.30 ± 56.55 ^ab^	1647.63 ± 39.82 ^ab^	1742.98 ± 32.76 ^b^	1690.33 ± 55.75 ^ab^
FI (g/bird)	1–21	1169.88 ± 18.03	1153.10 ± 10.46	1111.35 ± 30.52	1104.87 ± 37.66	1160.58 ± 20.14	1131.58 ± 10.33
	22–35	1748.38 ± 24.66	1761.45 ± 51.10	1650.32 ± 104.62	1653.68 ± 40.06	1740.27 ± 63.75	1760.85 ± 44.20
	1–35	2918.27 ± 38.40	2914.55 ± 55.02	2922.88 ± 158.00	2758.53 ± 51.98	2900.85 ± 70.40	2892.42 ± 53.92
FCR (feed/gain)	1–21	1.43 ± 0.17 ^c^	1.35 ± 0.17 ^a^	1.37 ± 0.15 ^ab^	1.40 ± 0.10 ^bc^	1.37 ± 0.11 ^ab^	1.34 ± 0.15 ^a^
	22–35	2.53 ± 0.12 ^b^	2.10 ± 0.08 ^a^	2.06 ± 0.06 ^a^	1.97 ± 0.06 ^a^	1.89 ± 0.04 ^a^	2.01 ± 0.07 ^a^
	1–35	1.87 ± 0.04 ^b^	1.68 ± 0.03 ^a^	1.65 ± 0.03 ^a^	1.61 ± 0.03 ^a^	1.60 ± 0.03 ^a^	1.61 ± 0.03 ^a^
Mortality rate (%)	1–21	2.08	2.08	2.08	NIL	NIL	2.08
	22–35	2.08	NIL	2.08	NIL	2.08	NIL
	1–35	4.17	2.08	4.17	NIL	2.08	2.08

Each value is the mean ± SE of six replicate cages with eight chickens each. ^a,b,c^ Means within the same row with different superscript letters differ significantly (*p* < 0.05). ^†^ C = control (basal diet); 1ϕ = BD + 1 g/kg phage cocktail; 2ϕ = BD + 2 g/kg phage cocktail; P = BD + 1 g/kg probiotic; 1ϕP = BD + 1 g/kg phage cocktail + 1 g/kg probiotic; 2ϕP = BD + 2 g/kg phage cocktail + 1 g/kg probiotic; BW = body weight; BWG = body weight gain.

**Table 4:** Structure of gut microbiota supplemented with different dietary treatments in the ilea of 35 day old chickens according to PERMANOVA (**a**) marginal and (**b**) pairwise test of Bray–Curtis similarities. The test includes the degrees of freedom (Df), sum of squares (SS), mean square (MS), and *p*-value under Monte Carlo correction (*p*_MC_).

a. Marginal Test					
Source	Df	SS	MS	Pseudo-F	*p*_MC_
Treatment	5	28,910	5782.1	4.0189	0.001
Residual	29	41,723	1438.7		
Total	34	70,634			
**b. Pairwise Test**					
**Groups ^†^**	**t**		**Unique Perms**	***p***_**MC**_	
1ϕ, 1ϕP	2.2179		403	0.011	
1ϕ, 2ϕ	0.99346		405	0.419	
1ϕ, 2ϕP	2.7819		405	0.002	
1ϕ, C	0.97549		408	0.437	
1ϕ, P	0.78783		413	0.672	
1ϕP, 2ϕ	2.6116		401	0.002	
1ϕP,2ϕP	1.179		418	0.234	
1ϕP, C	2.4102		403	0.006	
1ϕP, P	2.2141		410	0.005	
2ϕ, 2ϕP	3.2675		401	0.002	
2ϕ, C	0.80525		408	0.651	
2ϕ, P	0.98786		397	0.393	
2ϕP, C	3.167		399	0.001	
2ϕP, P	2.7201		400	0.002	
C, P	1.0209		394	0.3850	

^†^ Treatments: C = control (basal diet); 1ϕ = BD + 1 g/kg phage cocktail; 2ϕ = BD + 2 g/kg phage cocktail; P = BD + 1 g/kg probiotics; 1ϕP = BD + 1 g/kg phage cocktail + 1 g/kg probiotics; 2ϕP = BD + 2 g/kg phage cocktail + 1 g/kg probiotics.

**Table 5:** List of OTUs that were significantly higher in ϕP than in non-ϕP groups.

OTUs	Taxonomy	LogFC	Standard Error (SE)	*p*-Values	Adjusted *p*-Values
Otu000006	*Bacteroides uniformis*	3.238460356	0.513486	2.85 ×10^−10^	1.64 ×10^−8^
Otu000001	*Bacteroides*	3.082429046	0.511617	1.69 ×10^−9^	6.49 ×10^−8^
Otu000585	*Odoribacter*	2.884646375	1.034084	0.005278	0.02529
Otu000009	*Alistipes*	2.764961394	0.432556	1.64 ×10^−10^	1.64 ×10^−8^
Otu000139	*Alistipes finegoldii*	2.723944494	0.680663	6.28 ×10^−5^	0.000723
Otu000269	*Ruminococcaceae_unclassified*	2.720698332	1.02679	0.008056	0.034313
Otu000070	*Alistipes*	2.647180277	0.526343	4.92 ×10^−7^	1.41 ×10^−5^
Otu000624	*Ruminococcaceae UCG-014*	2.618442967	1.056871	0.013229	0.04612
Otu000111	*Anaerotruncus*	2.539148885	0.862369	0.003236	0.018607
Otu000014	*Ruminococcus*	2.495897314	0.525048	2.00 ×10^−6^	4.59 ×10^−5^
Otu000031	*Lachnospiraceae_unclassified*	2.470502349	0.558793	9.82 ×10^−6^	0.000188
Otu000117	*Ruminococcaceae UCG-005*	2.445622307	0.891623	0.00609	0.028015
Otu000048	*Bacillaceae_unclassified*	2.308772376	0.862055	0.007401	0.032737
Otu000170	*Bacteroides*	2.245170744	0.57669	9.89 ×10^−5^	0.001034
Otu000319	*Rhodospirillaceae*	2.162299849	0.88805	0.014897	0.047586
Otu000005	*Alistipes onderdonkii*	2.121163741	0.521993	4.83 ×10^−5^	0.000695
Otu000210	*Anaerotruncus*	2.025946607	0.945538	0.032142	0.080355
Otu000015	*Clostridium X1Vb*	2.019893556	0.617525	0.001072	0.007704
Otu000337	*Desulfovibrio*	2.014867537	0.841755	0.016682	0.049954
Otu000675	*Anaerostipes*	2.013275931	1.087486	0.064125	0.122906
Otu000032	*Clostridium X1Va*	1.982675782	0.536143	0.000217	0.002082
Otu000643	*Vampirovibrio*	1.967914419	1.072608	0.06655	0.125463
Otu000353	*Alistipes putredinis*	1.899173634	0.770005	0.013646	0.046157
Otu000204	*Ruminococcaceae UCG-014*	1.859164848	0.750237	0.013208	0.04612
Otu000157	*Ruminococcaceae_unclassified*	1.841791976	0.706923	0.009178	0.036394
Otu000866	*Clostridium*	1.840566564	0.885046	0.03756	0.086387
Otu000367	*Coprobacillus*	1.822128533	1.039682	0.079674	0.143164
Otu000694	*Lachnospiraceae_unclassified*	1.815009851	1.076386	0.091756	0.158407
Otu000066	*Bacteroides fragilis*	1.803001647	0.592086	0.002325	0.014857
Otu000025	*Lachnospiraceae_unclassified*	1.784599464	0.409758	1.33 ×10^−5^	0.000218
Otu000426	*Ruminococcaceae UCG-014*	1.782185523	0.793533	0.024711	0.063151
Otu000027	*Eisenbergiella*	1.766953157	0.495082	0.000358	0.00317
Otu000067	*Butyricimonas*	1.753087241	0.611499	0.004146	0.02165
Otu000075	*Lachnospiraceae_unclassified*	1.747367694	0.612453	0.00433	0.02165
Otu000247	*Bacteria_unclassified*	1.741509274	0.770742	0.023851	0.062338
Otu000022	*Faecalibacterium prausnitzii*	1.738699859	0.433282	6.00 ×10^−5^	0.000723
Otu000104	*Lachnospiraceae_unclassified*	1.715040783	0.493786	0.000514	0.004224
Otu002794	*Lachnospiraceae_unclassified*	1.702047151	1.080272	0.115124	0.194695
Otu000095	*Oscillospira*	1.686705907	0.725797	0.020129	0.055115
Otu000338	*Ruminococcaceae UCG-014*	1.677289282	0.969175	0.083517	0.14776
Otu000186	*Anaerotruncus*	1.668325958	0.655373	0.010909	0.041816
Otu000148	*Oscillospira*	1.665393273	0.699204	0.017226	0.049954
Otu000947	*Clostridiales*	1.659154189	1.306282	0.204037	0.312856
Otu000802	*Clostridium IV*	1.654944242	0.813506	0.041918	0.090954
Otu000041	*Clostridium X1Va*	1.644739429	0.517848	0.001493	0.010098
Otu000069	*Ruminococcus*	1.601694881	0.542143	0.003133	0.018607
Otu000044	*Lachnospiraceae_unclassified*	1.575177456	0.551342	0.004277	0.02165
Otu000573	*Ruminococcus*	1.546199668	1.1055	0.16192	0.258623
Otu000057	*Ruminococcus*	1.517227727	0.458007	0.000924	0.007085
Otu000007	*Phascolarctobacterium*	1.505713845	0.607781	0.013234	0.04612

Note: LogFC is directly related to abundance in the ϕP group, where a higher number denotes a higher abundance. Phage cocktail and probiotic combination groups (ϕP): 1ϕP and 2ϕP; other groups (non-ϕP): C, 1ϕ, 2ϕ, and P.

## Data Availability

Not applicable.

## Supplementary Materials

The following supporting information can be downloaded at: https://www.mdpi.com/article/10.3390/ani13081328/s1, Figure S1: Rarefaction curves of species (OTUs) versus sample size (number of sequences) plotted at 97% sequences identity; Figure S2: The spread of gut microbiota of different age (21 and 35 d) of chickens investigated based on Principal coordinate analysis (PCO) of Bray-Curtis similarity index; Figure S3: The spread of gut microbiota of different part of intestine (ilea and caeca) of chickens investigated based on Principal coordinate analysis (PCO) of Bray-Curtis similarity index; Figure S4: Structure of gut microbiota supplemented with different dietary treatments in ilea and caeca of 21 and 35 d chickens investigated based on Principal coordinate analysis(PCO) of Bray-Curtis similarity index; Figure S5: Structure of gut microbiota supplemented with different dietary treatments in ilea of 21 and 35 d chickens investigated based on Canonical analysis of principal coordinates (CAP) of Bray-Curtis similarity index; Table S1: Alpha diversity of measured number of observed OTUs, ACE, Shannon and Inverse Simpson from sequences that were normalised to 9998; Table S2: PERMANOVA marginal test on Bray-Curtis similarities (includes degrees of freedom (Df), sum of squares (SS), mean square (MS) and P value under Monte-Carlo correction (PMC)) for gut microbiota diversity; Table S3: Structure of gut microbiota supplemented with different dietary treatments in ilea of 21 and 35 d old chickens based on PERMANOVA (a) marginal and (b) pairwise test of Bray-Curtis similarities.
