# Supplementary material for: Effects of Phage Cocktail, Probiotics, and Their Combination on Growth Performance and Gut Microbiota of Broiler Chickens
Source: Animals (Basel). 2023 Apr 13;13(8):1328. doi: 10.3390/ani13081328 (PMC10135209; doi:10.3390/ani13081328)
Supplement: Supplementary file 1 [file animals-13-01328-s001.zip › animals-2209422-supplementary.pdf]

Supplementary data

Alpha diversity profiles of gut microbiota

The final filtered and processed sequences were comprised of 3,404,883 sequences reads with a mean length of 464 bp. Among them, 153,643 reads were unique sequences. Furthermore, a total of 16,381 OTUs were obtained after the sequences were clustered based on 97% homology cut-off. The resulting dataset that was normalised to 9998 sequences, registered an average of 97.3% sequence coverage (Table S1, Figure S1). This suggests that the depth of sequencing applied in this study covered most of the bacterial populations in the samples. There was no difference in bacterial richness (ACE) between control and treatment groups in 21 d ilea, and 21 d and 35 d caeca (Table S1). This was not the case in 21 d ilea where the bacterial richness was significantly lower between control and treatment groups, except in P group. There was no difference in bacterial diversity (Shannon) between control and treatment groups in 21 d ilea and caeca, and 35 d caeca. The 2ϕP of 35 d ilea group had significantly higher bacterial diversity than the control group.

Table S1 Alpha diversity of measured number of observed OTUs, ACE, Shannon and Inverse Simpson from sequences that were normalised to 9998.

Group	Sobs	Coverage	ACE	Shannon	Inverse Simpson
21I_C	562.17 ± 26.81 ^{cde}	0.973 ± 0.001 ^{a}	1018.91 ± 86.10 ^{g}	3.47 ± 0.09 ^{abcde}	11.76 ± 1.43 ^{abc}
21I_1ϕ	550.50 ± 20.29 ^{cde}	0.978 ± 0.003 ^{abc}	811.77 ± 70.43 ^{def}	3.43 ± 0.08 ^{abcde}	9.74 ± 1.10 ^{abc}
21I_2ϕ	496.83 ± 37.30 ^{bc}	0.982 ± 0.002 ^{bc}	683.37 ± 63.29 ^{cd}	3.28 ± 0.22 ^{abc}	10.10 ± 2.24 ^{abc}
21I_P	567.83 ± 20.94 ^{cde}	0.976 ± 0.002 ^{ab}	868.52 ± 46.20 ^{defg}	3.49 ± 0.15 ^{abcde}	11.47 ± 1.75 ^{abc}
21I_1ϕP	501.50 ± 33.03 ^{bc}	0.982 ± 0.003 ^{bc}	712.75 ± 79.78 ^{cde}	3.52 ± 0.22 ^{abcde}	13.52 ± 3.11 ^{abc}
21I_2ϕP	437.17 ± 18.93 ^{b}	0.982 ± 0.002 ^{bc}	709.14 ± 68.39 ^{cde}	3.06 ± 0.19 ^{ab}	8.18 ± 1.65 ^{abc}
35I_C	503.67 ± 40.89 ^{bc}	0.983 ± 0.003 ^{c}	734.80 ± 107.38 ^{cdef}	3.33 ± 0.20 ^{abcd}	9.46 ± 1.96 ^{abc}
35I_1ϕ	620.40 ± 146.55 ^{ef}	0.979 ± 0.002 ^{bc}	817.34 ± 80.40 ^{def}	3.64 ± 0.19 ^{cde}	11.28 ± 1.87 ^{abc}
35I_2ϕ	611.80 ± 33.81 ^{def}	0.976 ± 0.003 ^{ab}	890.51 ± 82.10 ^{efg}	3.44 ± 0.21 ^{abcde}	9.27 ± 1.91 ^{abc}
35I_P	691.33 ± 78.00 ^{f}	0.978 ± 0.004 ^{abc}	896.65 ± 113.08 ^{efg}	3.88 ± 0.16 ^{de}	13.86 ± 2.58 ^{bc}
35I_1ϕP	694.60 ± 36.49 ^{f}	0.978 ± 0.001 ^{abc}	905.07 ± 30.98 ^{fg}	3.87 ± 0.08 ^{de}	11.73 ± 1.03 ^{abc}
35I_2ϕP	514.83 ± 26.48 ^{bcd}	0.990 ± 0.001 ^{d}	586.11 ± 45.91 ^{bc}	3.91 ± 0.10 ^{e}	15.41 ± 1.73 ^{c}
21C_C	306.67 ± 9.13 ^{a}	0.992 ± 0.001 ^{d}	383.20 ± 19.06 ^{a}	3.35 ± 0.09 ^{abcde}	9.24 ± 1.42 ^{abc}
21C_1ϕ	339.50 ± 19.31 ^{a}	0.992 ± 0.001 ^{d}	413.89 ± 24.88 ^{ab}	3.44 ± 0.13 ^{abcde}	10.94 ± 3.32 ^{abc}
21C_2ϕ	334.00 ± 24.25 ^{a}	0.993 ± 0.001 ^{d}	418.19 ± 32.44 ^{ab}	3.36 ± 0.18 ^{abcde}	10.55 ± 3.46 ^{abc}
21C_P	344.00 ± 21.64 ^{a}	0.992 ± 0.001 ^{d}	432.26 ± 18.66 ^{ab}	3.56 ± 0.20 ^{bcde}	13.16 ± 2.47 ^{abc}
21C_1ϕP	334.33 ± 19.54 ^{a}	0.992 ± 0.001 ^{d}	399.60 ± 27.01 ^{ab}	3.35 ± 0.18 ^{abcde}	8.80 ± 1.30 ^{abc}
21C_2ϕP	307.50 ± 24.22 ^{a}	0.993 ± 0.001 ^{d}	399.60 ± 27.23 ^{a}	3.35 ± 0.18 ^{abc}	8.80 ± 1.58 ^{ab}
35C_C	299.75 ± 31.80 ^{a}	0.995 ± 0.003 ^{d}	340.26 ± 54.14 ^{a}	3.46 ± 0.21 ^{abcde}	13.13 ± 4.83 ^{abc}
35C_1ϕ	323.17 ± 8.29 ^{a}	0.992 ± 0.001 ^{d}	388.56 ± 13.29 ^{a}	3.38 ± 0.10 ^{abcde}	8.15 ± 1.62 ^{abc}
35C_2ϕ	297.33 ± 7.14 ^{a}	0.994 ± 0.000 ^{d}	345.39 ± 7.26 ^{a}	3.21 ± 0.13 ^{abc}	7.05 ± 1.44 ^{ab}
35C_P	340.67 ± 18.68 ^{a}	0.992 ± 0.001 ^{d}	405.73 ± 26.97 ^{ab}	3.31 ± 0.16 ^{abcd}	9.66 ± 1.91 ^{abc}
35C_1ϕP	342.50 ± 19.01 ^{a}	0.992 ± 0.002 ^{d}	410.87 ± 33.72 ^{ab}	3.60 ± 0.12 ^{bcde}	12.62 ± 2.77 ^{abc}
35C_2ϕP	314.00 ± 14.76 ^{a}	0.993 ± 0.000 ^{d}	365.88 ± 13.62 ^{a}	2.99 ± 0.25 ^{a}	5.88 ± 2.02 ^{a}

Each value is mean ± SE of 6 replicate cages with 1 chicken each.
^{a,b,c,d,e,f,g}Means within the same column that have different superscripts differ significantly ($P < 0.05$).
For treatment (C = control (basal diet); 1ϕ = BD + 1 g/kg phage cocktail; 2ϕ = BD + 2 g/kg phage cocktail; P = BD + 1 g/kg probiotic; 1ϕP = BD + 1 g/kg phage cocktail + 1 g/kg probiotic; 2ϕP = BD + 2 g/kg phage cocktail + 1 g/kg probiotic), age (21 = 21-day-old; 35 = 35-day-old) and part of intestine (I = ilea, C = caeca). Sobs = number of observed OTUs.

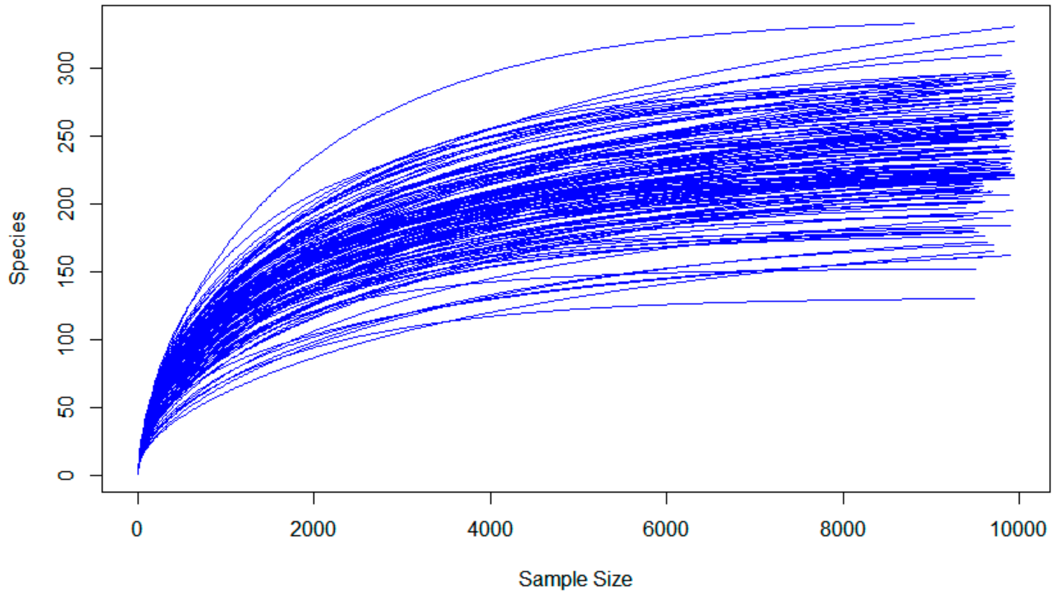

Figure S1 Rarefaction curves of species (OTUs) versus sample size (number of sequences) plotted at 97% sequences identity

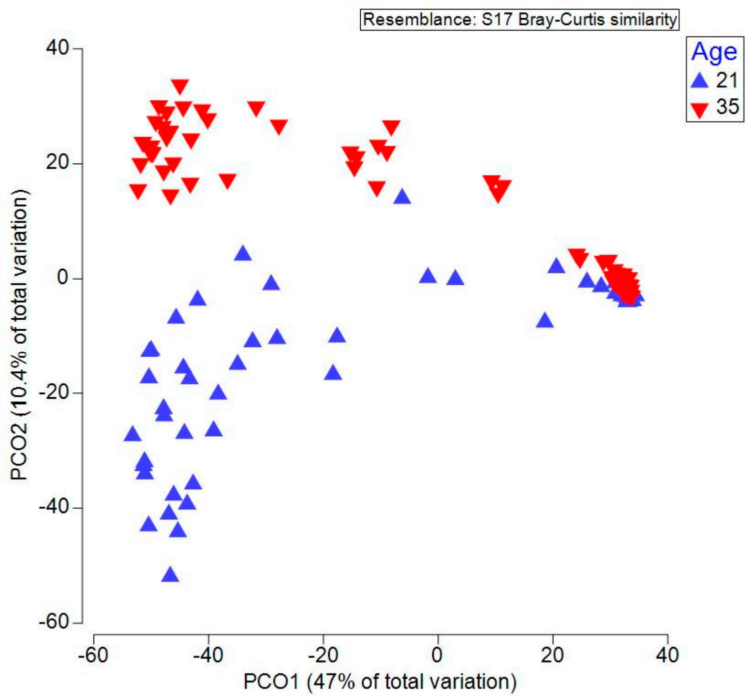

Figure S2 The spread of gut microbiota of different age (21 and 35 d) of chickens investigated based on Principal coordinate analysis (PCO) of Bray-Curtis similarity index

For age (21 = 21-day-old; 35 = 35-day-old).

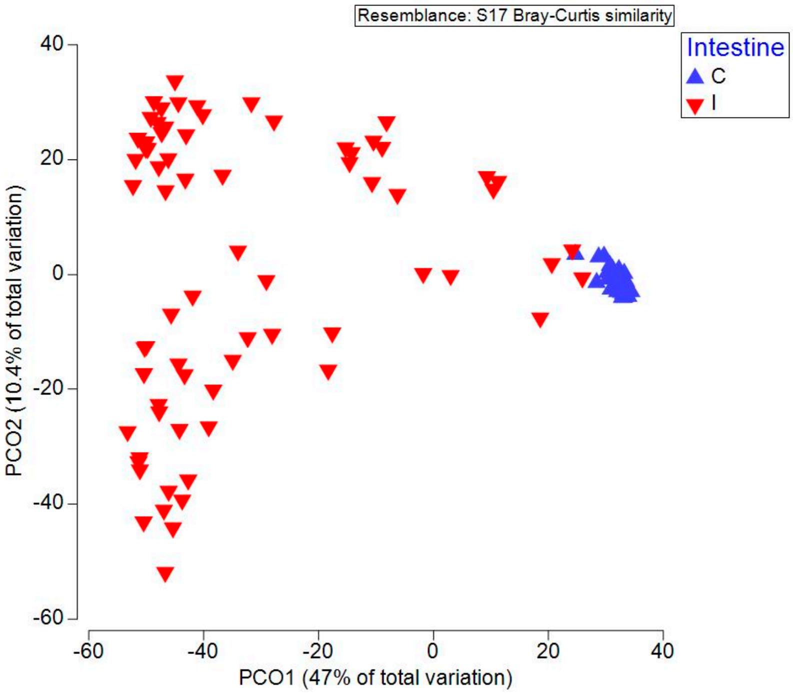

Figure S3 The spread of gut microbiota of different part of intestine (ilea and caeca) of chickens investigated based on Principal coordinate analysis (PCO) of Bray-Curtis similarity index

For part of intestine (I = ilea, C = caeca).

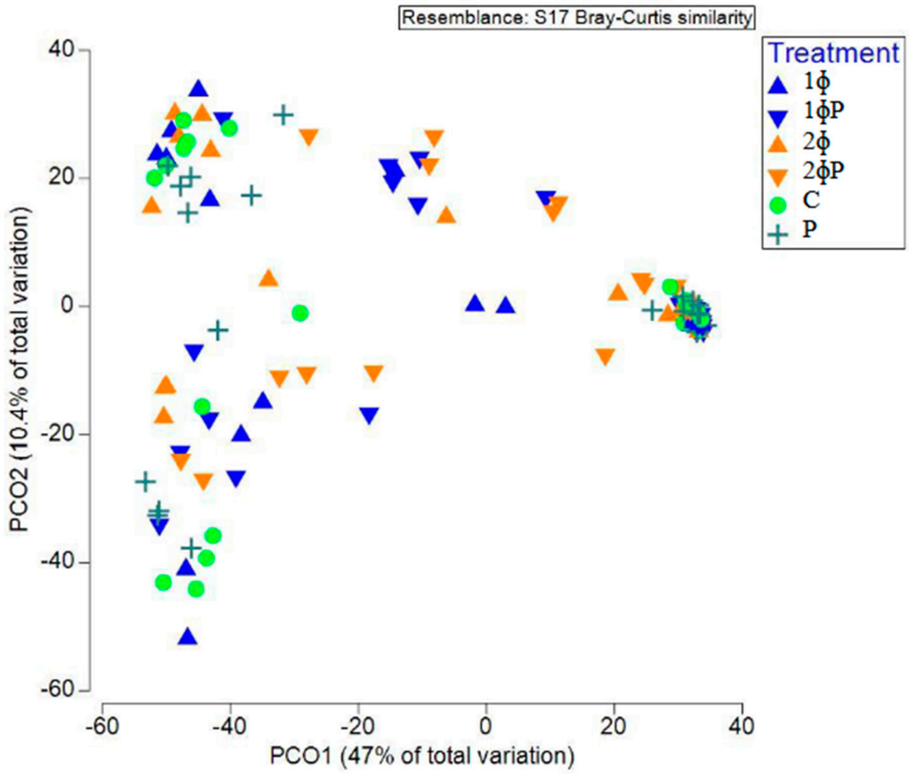

Figure S4 Structure of gut microbiota supplemented with different dietary treatments in ilea and caeca of 21 and 35 d chickens investigated based on Principal coordinate analysis(PCO) of Bray-Curtis similarity index. For treatment (C = control (basal diet); 1ϕ = BD + 1 g/kg phage cocktail; 2ϕ = BD + 2 g/kg phage cocktail; P = BD + 1 g/kg probiotic; 1ϕP =BD + 1 g/kg phage cocktail + 1 g/kg probiotic; 2ϕP = BD + 2 g/kg phage cocktail + 1 g/kg probiotic).

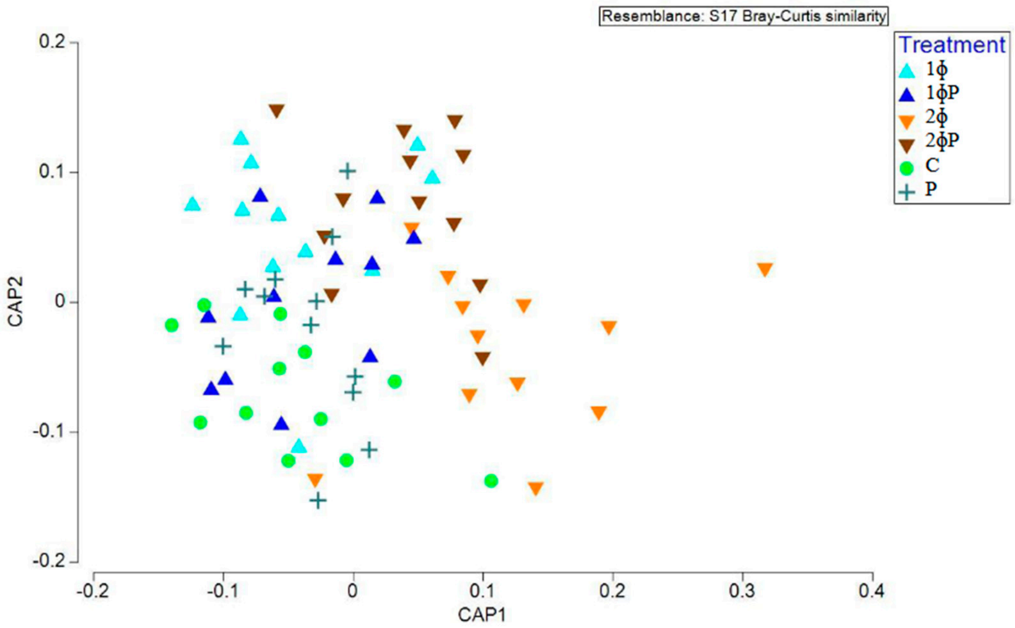

Figure S5. Structure of gut microbiota supplemented with different dietary treatments in ilea of 21 and 35 d chickens investigated based on Canonical analysis of principal coordinates (CAP) of Bray-Curtis similarity index.For treatment (C = control (basal diet); 1ϕ = BD + 1 g/kg phage cocktail; 2ϕ = BD + 2 g/kg phage cocktail; P = BD + 1 g/kg probiotic; 1ϕP =BD + 1 g/kg phage cocktail + 1 g/kg probiotic; 2ϕP = BD + 2 g/kg phage cocktail + 1 g/kg probiotic).

Table S2 PERMANOVA marginal test on Bray-Curtis similarities (includes degrees of freedom (Df), sum of squares (SS), mean square (MS) and P value under Monte-Carlo correction (P_{MC})) for gut microbiota diversity based on:

a. chicken age (21 d and 35 d).

Source	Df	SS	MS	Pseudo-F	P_{MC}
Age	1	23171	23171	8.8451	0.002
Residual	141	3.6937E+05	2619.7		
Total	142	3.9254E+05			

b. chicken intestine (ilea and caeca).

Source	Df	SS	MS	Pseudo-F	P_{MC}
Intestine	1	1.5383E+05	1.5383E+05	90.862	0.001
Residual	141	2.3871E+05	1693		
Total	142	3.9254E+05			

c. treatment.

Source	Df	SS	MS	Pseudo-F	P_{MC}
Treatment	5	13336	2667.1	0.96358	0.499
Residual	137	3.7921E+05	2767.9		
Total	142	3.9254E+05			

d. chicken caecum.

Source	Df	SS	MS	Pseudo-F	P_{MC}
Treatment	5	3267.1	653.42	0.91688	0.646
Residual	66	47035	712.65		
Total	71	50302			

e. 21 d chicken.

Source	Df	SS	MS	Pseudo-F	P_{MC}
Treatment	5	10415	2083	0.74755	0.727
Residual	66	1.8391E+05	2786.5		
Total	71	1.9432E+05			

f. ilea of 21 d chicken.

Source	Df	SS	MS	Pseudo-F	P_{MC}
Treatment	5	16426	3285.3	1.428	0.071
Residual	30	69017	2300.6		
Total	35	85444			

g. caeca of 21 d chicken.

	Df	SS	MS	Pseudo-F	P_{MC}
Treatment	5	3300	660	1.1654	0.134
Residual	30	16990	566.34		
Total	35	20290			

h. 35 d chicken.

Source	Df	SS	MS	Pseudo-F	P_{MC}
Treatment	5	15236	3047.2	1.2393	0.241

Residual	65	1.5982E+05	2458.7		
Total	70	1.7505E+05			

i. caeca of 35 d chicken.

Source	Df	SS	MS	Pseudo-F	P_{MC}
Treatment	5	2938.9	587.78	1.1231	0.22
Residual	30	15701	523.35		
Total	35	18639			

Table S3 Structure of gut microbiota supplemented with different dietary treatments in ilea of 21 and 35 d old chickens based on PERMANOVA (a) marginal and (b) pairwise test of Bray-Curtis similarities. The test includes degrees of freedom (Df), sum of squares (SS), mean square (MS) and *P value* under Monte-Carlo correction (P_{MC}).

a. Marginal test

Source	Df	SS	MS	Pseudo-F	P_{MC}
Treatment	5	23528	4705.6	1.855	0.005
Residual	65	1.6488E+05	2536.7		
Total	70	1.8841E+05			

b. Pairwise test

Groups†	t	Unique perms	P_{MC}
1ϕ, 1ϕP	1.0853	998	0.299
1ϕ, 2ϕ	0.73964	998	0.81
1ϕ, 2ϕP	1.5589	998	0.043
1ϕ, C	0.96796	999	0.457
1ϕ, P	0.66535	997	0.867
1ϕP, 2ϕ	1.2802	998	0.137
1ϕP, 2ϕP	1.1864	999	0.179
1ϕP, C	1.6169	996	0.026
1ϕP, P	1.4116	998	0.051
2ϕ, 2ϕP	1.9504	998	0.004
2ϕ, C	1.0898	999	0.325
2ϕ, P	0.86084	998	0.595
2ϕP, C	2.2436	999	0.001
2ϕP, P	2.0671	999	0.002
C, P	0.73884	999	0.804

†For treatment (C = control (basal diet); 1Φ = BD + 1 g/kg phage cocktail; 2Φ = BD + 2 g/kg phage cocktail; P = BD + 1 g/kg probiotic; 1ΦP = BD + 1 g/kg phage cocktail + 1 g/kg probiotic; 2ΦP = BD + 2 g/kg phage cocktail + 1 g/kg probiotic).
